# Postovulatory Aging of Mouse Oocytes Impairs Offspring Behavior by Causing Oxidative Stress and Damaging Mitochondria

**DOI:** 10.3390/cells13090758

**Published:** 2024-04-28

**Authors:** Ming-Tao Xu, Min Zhang, Guo-Liang Wang, Shuai Gong, Ming-Jiu Luo, Jie Zhang, Hong-Jie Yuan, Jing-He Tan

**Affiliations:** College of Animal Science and Veterinary Medicine, Shandong Agricultural University, Tai’an 271018, China; xumingtao321@126.com (M.-T.X.); mrzhangmin@163.com (M.Z.); 18763820795@163.com (G.-L.W.); gongshuai5@sdau.edu.cn (S.G.); luomj@sdau.edu.cn (M.-J.L.); zhuji29@sdau.edu.cn (J.Z.)

**Keywords:** mental disorder, mitochondrial dysfunction, offspring behavior, oxidative stress, postovulatory oocyte aging

## Abstract

Information on long-term effects of postovulatory oocyte aging (POA) on offspring is limited. Whether POA affects offspring by causing oxidative stress (OS) and mitochondrial damage is unknown. Here, in vivo-aged (IVA) mouse oocytes were collected 9 h after ovulation, while in vitro-aged (ITA) oocytes were obtained by culturing freshly ovulated oocytes for 9 h in media with low, moderate, or high antioxidant potential. Oocytes were fertilized in vitro and blastocysts transferred to produce F1 offspring. F1 mice were mated with naturally bred mice to generate F2 offspring. Both IVA and the ITA groups in low antioxidant medium showed significantly increased anxiety-like behavior and impaired spatial and fear learning/memory and hippocampal expression of anxiolytic and learning/memory-beneficial genes in both male and female F1 offspring. Furthermore, the aging in both groups increased OS and impaired mitochondrial function in oocytes, blastocysts, and hippocampus of F1 offspring; however, it did not affect the behavior of F2 offspring. It is concluded that POA caused OS and damaged mitochondria in aged oocytes, leading to defects in anxiety-like behavior and learning/memory of F1 offspring. Thus, POA is a crucial factor that causes psychological problems in offspring, and antioxidant measures may be taken to ameliorate the detrimental effects of POA on offspring.

## 1. Introduction

If not fertilized or activated in time after ovulation, oocytes of mammals undergo a time-dependent process of postovulatory oocyte aging (POA). Because some animals potentially undertake sexual activity on any day of the estrous cycle due to a lack of synchronization mechanisms between sexual activity and ovulation, fertilization may occur in aged oocytes. Many studies have demonstrated that the fertilization of aged oocytes can affect embryo development [1], cause abnormalities, and reduce longevity in offspring [2,3]. In human beings, POA might increase early pregnancy loss [4] and cause menstrual disorder and decreased fecundity in daughters [5]. Furthermore, POA has been proposed as one of the major causes of the decreased population of some endangered mammalian species [6]. Thus, the mechanisms for POA have been a hot area of research in recent years as there is an urgent need for its control.

However, most of the studies on POA reported thus far have focused on its effects on oocyte quality and early embryo development, and information about its long-term effects on offspring are limited. Tarín et al. [2] reported that insemination of aged mouse oocytes led to delayed development of the righting reflex and higher spontaneous motor activity and emotionality of offspring. However, the effects of POA on offspring’s anxiety behavior has not been reported, and its effect on offspring’s learning ability is still uncertain [2]. Furthermore, the mechanisms by which POA affects offspring are largely unknown.

Numerous studies have observed an increased oxidative stress (OS) during POA in different species, including mice [7], pigs [8], and bovines [9]. By analyzing the relationship between biochemical changes and the phenotypes in aging oocytes, Lord and Aitken [10] proposed that OS may be an initiator of a cascade of events that create the phenotypes of aged oocytes. Recent studies showed that supplementation with antioxidants could alleviate POA [9,11,12]. However, although this suggests indirectly that OS might deteriorate POA oocytes, direct evidence for a cause–effect relationship between OS and the phenotypes of aged oocytes is lacking. Furthermore, whether the long-term effects of POA on offspring are caused by increased OS is unknown.

Depression and anxiety disorders [13,14,15] and cognitive disorders such as Alzheimer’s disease [16,17] are among the major public health problems. However, mechanisms for the pathogenesis of these mental disorders are largely unclear. It is known that the mitochondria, which are inherited exclusively from the oocyte, are heavily implicated in learning, memory, cognition, and virtually every mental or neurological affliction [18]. Damaged mitochondria from oocytes can carry information that causes mitochondrial and metabolic dysfunction of the next generation [19]. Furthermore, during pathogenesis of the programmable metabolic syndrome, such as in type 2 diabetes, OS promotes epigenetic modifications of vital genes [20]. We thus hypothesized that POA might increase OS and damage mitochondria within oocytes, leading to impaired mitochondrial function and behavior in offspring.

The objective of the present study was to test this hypothesis by using both in vivo aging (IVA) and in vitro aging (ITA) with increased OS of mouse oocytes. While the IVA oocytes were collected at 9 h after ovulation with freshly ovulated oocytes used as controls, the ITA oocytes were obtained by culturing the freshly ovulated oocytes for 9 h in media with different antioxidant levels. Both IVA and ITA oocytes were fertilized in vitro, and the resultant blastocysts were transferred to pseudopregnant recipients. Anxiety-like behavior, as well as spatial and fear learning/memory and expression of related genes, were observed in F1 and F2 offspring. Furthermore, OS and mitochondrial function were examined in aged oocytes, blastocysts, and adult hippocampi to verify that the impaired mitochondrial function and OS in aged oocytes were passed on to the adult brain to affect offspring behavior.

## 2. Materials and Methods

We carried out the study according to the relevant guidelines and regulations. We conducted mouse care and handling strictly in accordance with the guidelines issued by the Animal Care and Use Committee of the Shandong Agricultural University, PR China (permit SDAUA-2019-004). We purchased all the chemicals and reagents used in this study from Sigma Chemical Co., St. Louis, MO, USA, unless mentioned otherwise.

### 2.1. Mouse Treatment and Oocyte Recovery

We used mice of the Kunming strain, which were originally derived from ICR (CD-1) mice. We raised the mice in a room under a 14 h light and 10 h darkness photoperiod, with lights-off at 20:00 h. We superovulated female mice (6–8 weeks of age) by intraperitoneal injection of 10 IU equine chorionic gonadotropin (eCG, Ningbo Hormone Product Co., Ltd., Ningbo, China) and 10 IU human chorionic gonadotropin (hCG, Ningbo Hormone Product Co., Ltd., Ningbo, China) at a 48 h interval. To recover freshly ovulated oocytes and the IVA oocytes, the superovulated mice were euthanized at 13 h and 22 h, respectively, after the hCG injection. Some of the superovulated mice were euthanized at 31 h following the hCG injection to collect the 18 h-aged oocytes. The oocytes were recovered by rupturing the oviduct ampullae in the M2 medium.

### 2.2. In Vitro Aging of Oocytes

To obtain ITA oocytes, the freshly ovulated oocytes recovered were washed in M2 and CZB medium, and then they were cultured for 9 h for aging in media with different levels of antioxidant potential. While the high-antioxidant medium consisted of CZB medium supplemented with 100 µM cysteamine and 200 µM cystine, the low-antioxidant medium consisted of CZB supplemented with 100 µM H_2_O_2_. The moderate-antioxidant medium consisted of the CZB medium alone.

### 2.3. In Vitro Fertilization and Embryo Culture

We used male mice at the age of 8–12 weeks. The mice were killed by cervical dislocation to collect cauda epididymis and vas deferens. We used the edge of an injection needle to cut the cauda epididymis and vas deferens several times in M2 medium before squeezing them and collecting sperm masses. Then, the sperm masses were put in a 1 mL drop of T6 medium with 10 mg/mL bovine serum albumin (BSA) and incubated for 15 min at 37 °C before the sperm suspension was collected and the sperm concentration measured. By using the same medium, the sperm concentration was adjusted to 2 to 4 × 10^7^ sperm/mL for capacitation, which was conducted at 37 °C for 1.5 h. Following washing in the fertilization medium (T6 containing 20 mg/mL BSA), about 30 oocytes were placed in a 150 μL drop of fertilization medium. Then, capacitated sperm were added to the fertilization drop to produce a final sperm concentration of about 1 × 10^6^ sperm/mL. Fertilization was observed at 6 h, and four-cell and blastocyst development was examined at 48 h and 96 h, respectively, after the insemination. To observe fertilization, cumulus cells and attaching spermatozoa were removed from oocytes, and the oocytes were fixed for 20 min in 4% paraformaldehyde. The fixed oocytes were stained for 5 min in M2 with 10 µg/mL Hoechst 33342, and observed for fertilization under a fluorescence microscope. Oocytes showing two pronuclei and the second polar body and/or first polar body were judged as fertilized in this study. To observe embryo development, at the end of the 6 h fertilization incubation, the inseminated oocytes were cultured in regular CZB medium, and after 48 culturing, the oocytes were transferred to CZB medium containing 5.55 mM glucose. Percentages of fertilized oocytes and four-cell and blastocyst embryos were calculated from inseminated oocytes.

### 2.4. Reactive Oxygen Species (ROS) Measurement in Oocytes and Blastocysts

We detected ROS levels in oocytes or blastocysts by measuring the H_2_O_2_ level. The H_2_O_2_ level was measured by staining with 2′,7′-dichloro-dihydro-fluorescein diacetate (DCHFDA). First, the 1-mM DCHFDA stock solution was diluted to 10 µM using M2 medium. Then, the resultant solution was used to stain cumulus-free oocytes/blastocysts at 37 °C for 10 min. At the end of the staining, the oocytes/blastocysts were washed in M2, placed on a slide, and observed under a Leica fluorescence microscope. The fluorescence was obtained by excitation at 488 nm. All the pictures were taken using fixed parameters of the microscope to ensure data consistency. The fluorescence intensity value of each oocyte/blastocyst was analyzed using Image-Pro Plus software (version 6.0.0.260).

### 2.5. Mitochondrial Membrane Potential (MMP) Measurement in Oocytes and Blastocysts

Detection of MMP was performed using an MMP detection (JC-1) kit (Beyotime Biotechnology Research Institute, Shanghai, China). Briefly, cumulus-free oocytes or blastocysts were washed thrice in M2 before being placed in a drop of working solution consisting of 100 µL M2 and 100 µL JC-1 dye. The oocytes/blastocysts were then cultured in working solution for 25 min at 37 °C. After being washed thrice in JC-1 staining buffer, the oocytes were observed under a laser scanning confocal microscope. The same oocyte/blastocyst was observed through the Cy3 channel (red fluorescence) and the FITC channel (green fluorescence). While detection of the aggregate JC-1 (red fluorescence) was done at 570 nm of emission wavelength, detection of the monomeric JC-1 (green fluorescence) was accomplished at 512 nm. The ratio of aggregated/monomeric JC-1 was calculated to represent the MMP level. A decrease in red/green JC-1 ratio indicated mitochondrial depolarization.

### 2.6. Measurement of Intraoocyte Glutathione

Spectrophotometric measurement for concentrations of total glutathione (GSX) and oxidized glutathione (GSSG) was performed using a commercial assay kit (Beyotime, S0053, Beyotime Institute of Biotechnology, Shanghai, China). Approximately 40 cumulus-free oocytes were mixed with 30 µL of protein scavenger and then vortexed for 5 min. After that, the mixture was frozen at −80 °C for 3 min and thawed thrice at room temperature. The mixture was centrifuged for 10 min at 4 °C and 10,000× *g*, and then the supernatant was recovered for measurement or stored at −80 °C before measurement. The 5,5′-dithiobis (2-nitrobenzoic acid) (DTNB)-GSSG reductase-recycling assay was carried out to measure the concentration of GSX. In addition, 0.5, 1, 2, 5, 10 and 15 µM GSX standards and sample blank with no GSX were measured. A spectrophotometer (Beckman Coulter DU 800) was used to obtain absorbance at 405 nm. The intra-oocyte concentration of GSX (pmol/oocyte) was calculated by dividing the GSX amount by the number of oocytes in each sample. Values of reduced glutathione (GSH) were obtained by calculating the difference between GSX and GSSG for each oocyte.

### 2.7. Embryo Transfer

To prepare recipients for embryo transfer, of 8- to 10-week-old female mice were mated with vasectomized males, and the mice that showed a copulatory plug the next morning (0.5 dpc) were used as pseudopregnant recipients. Because the IVA oocytes were inseminated 9 h later than the freshly ovulated oocytes, recipients of IVA oocyte-derived embryos were mated with vasectomized males one day later than the recipients for freshly ovulated oocyte-derived embryos. While transfer of the embryos from freshly ovulated oocytes was conducted at 18:00 on 2.5 dpc of the recipients, transfer of the embryos from IVA oocytes was performed at 07:00 on 2.5 dpc of the recipients. About 15 good-quality blastocysts were transferred to each recipient, with each uterine horn receiving 7 or 8 blastocysts. Transfer of embryos from the ITA oocytes followed the same procedures for transfer of embryos from the IVA oocytes.

### 2.8. Birth and Housing of F1 Offspring

Following embryo transfer, the recipients were caged singly until parturition. Starting from day 15 after embryo transfer, the recipients were checked for parturition 3 times a day (morning, noon and evening). Litter size and sex of the F1 pups were recorded within 12 h following parturition. On day 3 after parturition, the litter size and sex ratio of F1 pups per recipient were adjusted to 5–6 and 2:3, 3:2 or 3:3, respectively. The F1 offspring were weaned and weighed on day 21 after birth. The weaned male and female mice were caged separately before experiments. The naturally bred mice were produced from natural mating between males and non-superovulated females and the litter size and sex ratio of the pups were adjusted to 6 and 3:3, respectively.

### 2.9. Generation and Housing of F2 Offspring

To generate F2 offspring, one week after the behavioral tests, one male or female F1 mouse was taken from each litter and caged with a naturally bred female or male mouse, respectively. Seven days later, the female mice were caged singly until parturition. Starting from day 18 after caging, the females were checked for parturition 3 times a day (morning, noon and evening). Litter size and sex of the F2 pups were recorded within 12 h following parturition. On day 3 after birth, the litter size and sex ratio of F2 pups per litter were adjusted to 6 and 3:3, respectively. The F2 offspring were weaned and weighed on day 21 after birth. After weaning, the male and female mice were caged separately before experiments.

### 2.10. Behavioral Tests

Behavior was recorded via a video camera, which was mounted on the ceiling above the center of the test device and connected to an Any-maze video tracking motion analysis system (Stoelting, Wood Dale, IL, United States) operating on a personal computer. The temperature of the test room was kept constant (22–25 °C) and a white noise of 50–55 db was provided during tests. The tests were always carried out between 8:00 and 11:00 in the morning. Prior to each test, we placed the mice in the test room for 30 min to habituate them to the environment, and after each trial, we cleaned the device with 10% ethanol to remove the scent left by the previous animal.

The elevated plus maze test (EPM) was conducted when the offspring grew to 8 weeks old. The plus maze consisted of two open arms (30 × 6 cm) alternating at right angles with two closed arms (30 × 6 × 15 cm). The central platform delimited by the four arms was 36 cm^2^. The whole maze was elevated 50 cm above the floor. To start the EPM test, we placed a mouse in the central platform facing an open arm, and allowed it to explore the maze for 5 min. Following a four-paw criterion (when all four paws of the mouse enter a certain area, the software automatically records the data), numbers of entries and time spent in each arm during the total exploration in both open and closed arms were recorded and calculated using the Any-maze software (version 4.63).

The open-field test (OFT) was conducted three days after the EPM test. The open field consisted of a black square floor (50 × 50 cm) and white walls (45 cm high). The square floor was divided into 25 squares of 10 cm × 10 cm, with the 9 intermediate squares being the central area and the other 16 squares being the peripheral area. To start the OFT, we placed a mouse in the central area and allowed it to explore the open field for 5 min. The numbers of central entries, time spent in the central area, and distance traveled in the open field were recorded and calculated using the Any-maze software (version 4.63).

Three days after the OFT, a passive avoidance test (PAT) was performed using a PAT apparatus made by Shanghai Xinruan Information Technology Co., Ltd., Shanghai, China. The apparatus consisted of a light chamber and a dark chamber connected with a sliding door. The two chambers had a grid floor that can deliver an electric shock. The test consisted of two trials. In the acquisition trial, a mouse was placed in the light chamber, facing the dark chamber. As soon as the mouse entered the dark chamber, the door was closed and a foot shock (0.5 mA, 5 s) was delivered. Immediately after the shock, the mouse was placed back in its home cage. The retention trial was performed exactly 24 h after the acquisition trial. The mouse was again placed in the light chamber, but no electrical shock was given when it entered the dark chamber. The time to enter the dark chamber in the acquisition and the retention trial was recorded as latency. In the retention trial, the maximum latency that was allowed when a mouse did not enter the dark chamber was 300 s.

The Morris water maze (MWM) test was conducted 3 days after the PAT. We used a circular pool of 110 cm in diameter and 32 cm in depth. The circular water maze is divided into four quadrants by two perpendicular lines passing through the center of the circle, which are successively defined as the first, second, third and fourth quadrant. The division of the four quadrants is carried out in the software interface, there are no markers and obstacles in the water, and the mice can swim freely. The circular platform was positioned in the center of one of the quadrants 1 cm below the water surface. The mice cannot see the platform and need to find it by memory based on the reference object. Different shapes of paper are pasted on the inner walls of different quadrants as reference objects. During the test, the water temperature was maintained at 21 ± 1 °C. On the day before the trial, the mouse was allowed to swim for 1 min in the maze without a platform to habituate to the environment. During the trial, the mouse was gently placed in the pool with its head towards the pool wall and allowed 90 s to locate and climb onto the platform. When the mouse located the platform within 90 s, it was allowed to stay on it for 20 s before placing it back in its home cage. If a mouse could not find the platform within 90 s, we guided it to the platform and allowed it to remain there for 20 s before returning it to its home cage. The actual escape latency to reach the platform was recorded when a mouse was able to find the platform within 90 s, whereas the escape latency for the mouse that could not locate the platform within 90 s was recorded as 90 s. The experimental trials took place across 4 days. On each experimental day, the mouse was subjected to two sessions of a 90 s trial, and the mean time spent locating the platform was used as the measure of escape latency.

### 2.11. Measurement of Serum Cortisol

We euthanized mice at about 10 weeks after birth by decollation and collected trunk blood (approximately 1 mL) into ice-cooled centrifuge tubes. We always completed blood collection from different groups of mice within 20 s after their release from cages. To separate sera, we centrifuged the blood samples (1700× *g*, 10 min, 4 °C) 30 min after the blood collection. We stored the collected sera at −80 °C until hormone assay. Radioimmunoassay of serum cortisol concentrations was performed by the Central Hospital of Tai’an City using kits from Wei-Fang (3V) Bioengineering Co. Ltd., Weifang, China. The minimum detection level of the cortisol assay was 0.15 ng/mL, and intra- and inter-assay coefficients of variation (CV) were 5.3% and 6.7%, respectively.

### 2.12. Reverse-Transcription Quantitative Real-Time Polymerase Chain Reaction (RT-qPCR)

In this study, mRNA levels in hippocampi were assayed using the common RT-qPCR, while those in oocytes or blastocysts were analyzed using the one-step RT-qPCR. For the common RT-qPCR, hippocampi were collected at the same time when blood recovery was performed as described above. We performed homogenization of hippocampi using Trizol reagent to isolate total RNA. We resuspended the isolated RNA samples in diethyl pyrocarbonate-treated MilliQ water (DEPC-ddH_2_O) and digested them with RNase-free DNase I (Takara Biotechniques, Dalian, China). We spectroscopically quantified the purified RNA at 260 nm. We assessed the RNA purity and integrity by determining the A_260_/A_280_ ratio (1.8–2.0) and conducting electrophoresis in 1% agarose. We conducted reverse transcription using a PrimeScript RT reagent Kit (Takara RR047A), and RT-qPCR using a TB Green Premix Ex Taq (Takara, RR420A) on a Mx3005P real-time PCR instrument (Stratagene, Valencia, CA, USA). We used Normfinder (https://www.moma.dk/software/normfinder, accessed on 30 October 2022), an algorithm-based tool, to identify the most stable genes among the seven commonly used reference genes in hippocampus samples from 11 mice. The ranking of expression stability calculated by Normfinder in these genes analyzed was Gapdh > H2az1 = B2m > β-actin > Hprt > Ppib >Rplpd. Thus, we used Gapdh as the internal reference gene. We normalized gene expression to the internal control, and expressed the values relative to calibrator samples using the 2^−(∆∆CT)^ method. The gene-specific primers we used for real-time PCR are shown in Supplementary Table S1.

For one-step RT-qPCR, oocytes were denuded of cumulus cells by pipetting in M2 medium with 0.1% hyaluronidase. The cumulus-free oocytes were lysed to extract RNA using a commercial cell lysis kit (CellAmp Direct Prep Kit for RT-PCR and protein analysis, Takara, code 3733Q, Shiga, Japan). We preserved the lysate obtained at –80 °C before use. We conducted mRNA quantification using a One-Step TB Green PrimeScript Plus RT-PCR kit (Perfect Real Time, Takara, code RR096A). We used a 10 µL reaction volume for the amplification reaction (1 µL template, 2.2 µL RNase free ddH_2_O, 5 µL 2 × One Step TB Green RT-PCR Buffer 4, 0.6 µL Takara Ex Taq HS Mix, 0.2 µL PrimeScript Plus RTase Mix, 0.2 µL ROX Reference Dye II, and 0.4 µL each of forward and reverse primers (10 µM). The gene-specific primers used are listed in Table S1.

### 2.13. Immunofluorescence Microscopy of Mitophagy-Related Proteins in Oocytes

Oocytes were always washed thrice in M2 between treatments and incubation was performed at room temperature unless otherwise specified. Firstly, oocytes were fixed in 4.0% paraformaldehyde for 1 h, permeabilized with D-PBS containing 0.5% Triton X-100, and incubated in D-PBS containing 3.0% BSA for 1 h. Then, the oocytes were incubated overnight at 4 °C with anti-PINK1 (1:100; ab216144, Abcam, Cambridge, UK) or anti-PARKIN (1:100; Cat # 2132, Cell Signaling Technology, Danvers, MA, USA) diluted in blocking solution. Then, oocytes were incubated for 1 h with Cy3-conjugated goat anti-rabbit IgG (1:1000; 111-165-003; Jackson ImmunoResearch, West Grove, PA, USA) and stained with 10 µg/mL Hoechst 33342 for 5 min. Finally, oocytes were mounted onto slides and examined using a confocal microscope. To ensure accuracy of quantification, all pictures were taken with identical settings and on a single plane with maximum fluorescence intensity. Fluorescence intensities on the raw images were measured using Image-Pro Plus software (Media Cybernetics Inc., Rockville, MD, USA, version 6.0.0.260) under fixed thresholds across all slides. We measured both the fluorescence density and the area of the objects giving fluorescence, and calculated the mean relative intensity of fluorescence for each oocyte.

### 2.14. Mitochondrial Function Assays in Hippocampi

**ROS (H_2_O_2_) production**: To quantify the expression of ROS, H_2_O_2_ was measured in adult hippocampi of F1 offspring using a Beyotime Hydrogen Peroxide Assay Kit (S0038). We homogenized the hippocampal tissue and schizolysis (lysis) solution supplied with the kit at a ratio of 10 mg:100 µL. After centrifugation at 12,000× *g* for 5 min, we collected the supernatants for further tests. We carried out all the above operations on ice. Finally, we placed the test tubes containing 50 µL of supernatants and 100 µL of test solutions at room temperature for 30 min and measured them instantly using a spectrometer at a wavelength of 560 nm. We calculated the concentration of H_2_O_2_ released according to a standard concentration curve, which was produced from standard solutions during the identical experiments.

**Malondialdehyde (MDA)**: We collected hippocampal tissues from each group and placed them into an ice-cold RIPA lysis buffer (Beyotime, Shanghai, China) at a ratio of 10 mg:100 µL. We then homogenized the samples with a polytron homogenizer and centrifuged them at 10,000× *g* for 10 min. We recovered the supernatant and used a lipid peroxidation MDA assay kit (Beyotime, Shanghai, China) for MDA measurement. We followed the manufacturer’s instructions to carry out the assay. We established standard curves, and expressed MDA levels as nmol/mg weight.

**ATP production**: We measured the ATP levels in the hippocampus using a kit (S0026, Beyotime, Shanghai, China) according to the manufacturer’s instructions. Briefly, we lysed the hippocampal tissue with ATP lysis buffer, then centrifuged it at 12,000× *g* for 5 min at 4 °C. We mixed a total of 20 µL supernatant with 100 µL luciferase reagent in a lighttight microplate and measured the mixture by luminance. We normalized ATP levels to the weight of each sample, and expressed them as fold change of treated over control.

**Cytochrome C oxidase activity**: Samples and the schizolysis solution supplied by the Micro Mitochondrial Respiratory Chain Complex IV Activity Assay Kit (BC0945; Solarbio, Beijing, China) were homogenized at a ratio of 10 mg:100 µL. After centrifugation at 600× *g* for 10 min, the supernatants were collected and centrifuged at 11,000× *g* for 15 min. Supernatant was discarded to obtain mitochondria. The activity of cytochrome C oxidase was determined using the Micro Mitochondrial Respiratory Chain Complex IV Activity Assay Kit according to the manufacturer’s instructions. Briefly, mitochondrial homogenates were added to the respective reaction buffers. The reaction mixture was transferred to a prewarmed quartz cuvette and immediately put into a spectrophotometer. The absorbance of reaction mixture was measured at 550 nm for Complex IV. Mitochondrial complex activity was expressed as nmol/min/g weight.

### 2.15. Data Analysis

In this study, each treatment included at least three replicates. We analyzed data from the behavioral test using linear mixed models. The models analyzed the effect of fixed factors (IVA or ITA) on anxiety-like behavior or learning/memory of offspring based on the analysis of random factors (litter effect). We analyzed behavioral data after outliers deviating by more than two standard deviations from the mean were excluded. We analyzed other data using one-way ANOVA when each measure included more than two groups or using independent-sample *t* tests when each measure consisted of only two groups. We used the Duncan multiple-comparison test to locate differences during ANOVA. We used the software Statistics Package for Social Sciences (SPSS 20, SPSS, Inc., Armonk, NY, USA). We expressed all data as means ± SEM, and we considered a difference as significant only when the *p* value was less than 0.05.

## 3. Results

### 3.1. Effects of IVA on Developmental Potential, OS, and Mitochondria in Oocytes

**Figure 1:**
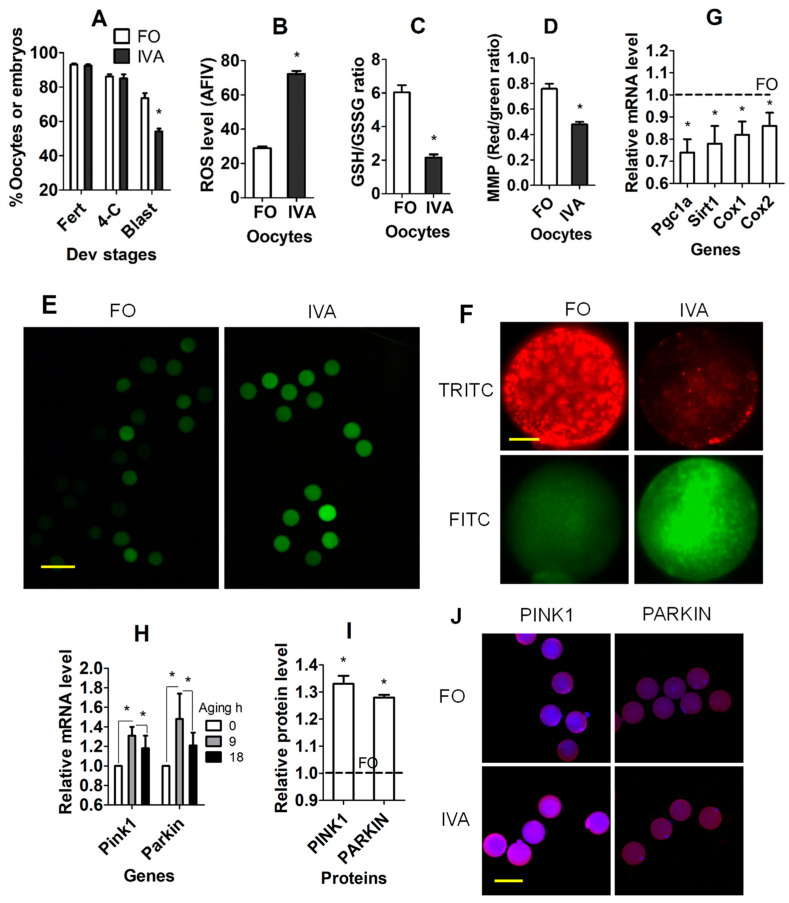
Effects of in vivo aging (IVA) on developmental potential, OS and mitochondria of oocytes. (**A**) Percentages of fertilized (Fert) oocytes and 4-cell (4-C) and blastocyst (Blast) embryos after fertilization of freshly ovulated (FO) and IVA oocytes. Each treatment was repeated 3 times, with each replicate containing 30–35 oocytes. (**B**–**D**,**G**,**I**) ROS level (average fluorescence intensity value, AFIV), reduced (GSH)/oxidized glutathione (GSSH) ratio, mitochondrial membrane potential (MMP, red/green ratio), mRNA level of mitochondrial function-related genes, and protein level of mitophagy-related genes (PINK1 and PARKIN), respectively, in FO and IVA oocytes. (**H**) mRNA levels of mitophagy-related genes in oocytes agedg for 0, 9 or 18 h in vivo. Each treatment was repeated 3 times, with each replicate containing about 30 (**B**), 40 (**C**), 25 (**D**), 40 (**G**,**H**) and 20–25 (**I**) oocytes. (**G**–**I**), mRNA/protein level of FO oocytes was set to one and that of IVA oocytes was expressed relative to one. * Significant difference (*p* < 0.05) from FO oocytes or between treatments. (**E**,**F**,**J**) Confocal images showing ROS, JC-1 and PINK1/PARKIN staining intensity, respectively, in FO and IVA oocytes. (**F**) Upper and lower images are of the same oocytes observed in TRITC (red fluorescence) and FITC (green) channels, respectively. Scale bars 120, 19, and 80 µm, in (**E**,**F**,**J**), respectively. Refer to Table S3-1 for numbers of oocytes used in different graphs.

Superovulated mice were euthanized at 13 and 22 h after hCG injection to recover freshly ovulated and 9 h-aged (IVA) oocytes, respectively. Immediately after recovery, oocytes were inseminated or examined for reactive oxygen species (ROS), reduced (GSH)/oxidized glutathione (GSSH) ratio, mitochondrial membrane potential (MMP), and the expression of mitochondria-related genes. Although percentages of fertilized oocytes and four-cell embryos did not differ between freshly ovulated and IVA oocytes, the percentage of blastocysts was significantly lower in IVA than in freshly ovulated oocytes (Figure 1A). While the level of ROS (Figure 1B,E) was significantly higher, levels of the GSH/GSSG ratio (Figure 1C) and MMP (Figure 1D,F) were significantly lower in IVA than in freshly ovulated oocytes. Furthermore, mRNA levels of gamma co-activator-1 alpha (Pgc1a), sirtuin 1 (Sirt1) and cytochrome C oxidase 1 (Cox1) and 2 (Cox2) genes were downregulated significantly in IVA compared to freshly ovulated oocytes (Figure 1G). Although mRNA levels of the mitophagy-related genes PTEN-induced kinase 1 (Pink1) and E3 ubiquitin protein ligase (Parkin) were increased in oocytes at 9 h of aging compared to freshly ovulated oocytes, they had decreased significantly by 18 h of aging (Figure 1H). Our immunocytochemistry also confirmed that the protein levels of PINK1 and PARKIN were higher in 9 h-aged oocytes than freshly ovulated oocytes (Figure 1I,J). The results suggest that (a) the oocytes that aged for 9 h in vivo can be fertilized successfully in vitro; (b) IVA for 9 h significantly increased OS and impaired mitochondrial function of oocytes; and (c) mitophagy increased during early aging as a response to the increase in damaged mitochondria, but it decreased in aged oocytes.

### 3.2. Litter Size and Sex Ratio at Birth in F1 Offspring from Freshly Ovulated or IVA Oocytes

Litter size and sex of the F1 pups were recorded within 12 h following parturition. Neither litter size nor the sex ratio was significantly different between F1 offspring from freshly ovulated oocytes and those from the 9 h-IVA oocytes (Table S2).

### 3.3. Effects of Oocyte IVA on Anxiety-Like Behavior and Blood Cortisol Level of F1 Offspring

**Figure 2:**
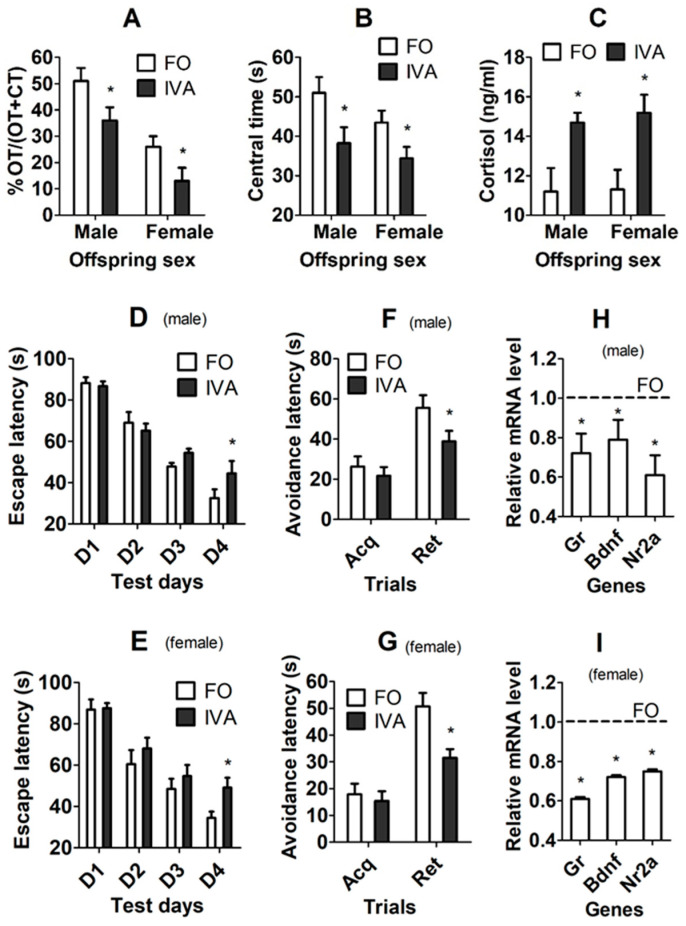
Effects of oocyte IVA on anxiety-like behavior and spatial/fear learning/memory in F1 offspring. (**A**–**I**) Open-arm time (OT)/(OT + closed arm time (CT)) of EPM, time (s) in central area of OFT, serum cortisol concentration, escape latency (s) on different days in MWM test, latency time (s) of acquisition (Acq) and retention (Ret) trials in PAT and mRNA levels of Gr, Bdnf and Nr2a genes in hippocampus, respectively, in F1 male and female offspring after transfer of embryos from freshly ovulated (FO) or IVA oocytes. (**H**,**I**) The value in FO offspring was set to one (dotted line) and the value in IVA offspring was expressed relative to one. For behavioral tests, each treatment contained 19–26 mice from 9–10 recipients. For cortisol measurement, each treatment was repeated 6–7 times, with each replicate containing serum from one mouse from a different recipient. For RT-qPCR assays, each treatment was repeated 6 times, with each replicate containing one mouse from a different litter. * Significant difference (*p* < 0.05) from FO offspring. Refer to Table S3-2 for numbers of mice used in different graphs.

The F1 offspring born from freshly ovulated or IVA oocytes were tested for anxiety-like behavior by elevated plus maze (EPM) and open-field test (OFT) and were measured for blood cortisol level by radioimmunoassay. In both male and female offspring, while open-arm time (OT)/OT + closed-arm time (CT) during EPM (Figure 2A) and the central area time during OFT (Figure 2B) were shorter, serum cortisol concentration (Figure 2C) was higher significantly in IVA offspring than in freshly ovulated oocyte-derived offspring. The results suggest that IVA significantly increased the anxiety-like behavior of both male and female F1 offspring.

### 3.4. Effects of IVA on Spatial/Fear Learning/Memory of F1 Offspring

A Morris water maze (MWM) test and a passive avoidance test (PAT) were performed to test the spatial and fear learning/memory of F1 offspring, respectively. In both male and female offspring, whereas the escape latency time was longer significantly on day 4 of the MWM test (Figure 2D,E), the avoidance latency time was shorter significantly on the retention trial of the PAT (Figure 2F,G) in IVA-derived offspring than in freshly ovulated oocyte-derived offspring. The results suggest that oocyte IVA significantly impaired the spatial/fear learning/memory of both male and female F1 offspring.

### 3.5. Effects of Oocyte IVA on mRNA Levels of Gr, Bdnf and Nr2a Genes in Hippocampi of F1 Offspring

To substantiate our above conclusions that oocyte IVA increases anxiety-like behavior and impairs spatial/fear learning/memory of offspring, expression of anxiety and learning/memory-related genes including glucocorticoid receptor (Gr), brain-derived neurotropic factor (Bdnf) and N-methyl-D-aspartate (NMDA) receptor subunit (Nr2a) were measured by RT-qPCR in hippocampi of the F1 offspring. The mRNA levels of all the three genes were significantly downregulated in both male and female IVA-derived offspring compared to those in freshly ovulated oocyte-derived offspring (Figure 2H,I). The results further confirmed that oocyte IVA downregulated hippocampal expression of the anxiolytic and learning/memory-beneficial genes in F1 offspring.

### 3.6. Comparison of Behaviors between F1 Offspring and Naturally Bred Mice

To determine the relationship between our F1 offspring from in vitro fertilization/embryo transfer and the naturally bred mice, behaviors were compared between F1 offspring from freshly ovulated oocytes and the age-matched naturally bred mice. The results showed no significant difference (*p* > 0.05) in either the open-arm time of the EPM test (Table S4-1), central area time in the OFT (Table S4-2), escape latency in the MWM test (Table S4-3), or latency time in the PAT (Table S4-4) between our F1 offspring and the naturally bred mice. The results suggest that (a) our superovulation, in vitro fertilization and embryo transfer procedures had no significant effects on behaviors of offspring and (b) POA impaired behaviors of F1 offspring in comparison with the naturally bred mice.

### 3.7. Effects of IVA on OS and Mitochondria in F1 Blastocysts

**Figure 3:**
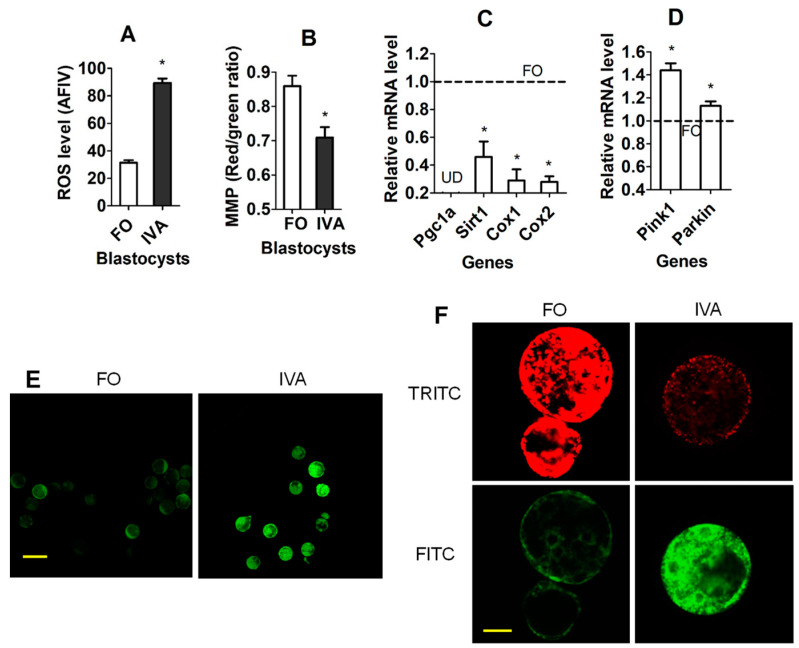
Effects of IVA on OS and mitochondria in F1 blastocysts. (**A**–**D**) Levels of ROS (AFIV), MMP (red/green ratio), mRNA of mitochondrial function- and mitophagy-related genes, respectively, in blastocysts from freshly ovulated oocytes (FO) and IVA oocytes. Each treatment was repeated 3 times, with each replicate containing 20–30 (**A**), 20 (**B**), and 40 (**C**,**D**) blastocysts. (**C**,**D**). The value in FO blastocysts was set to one (dotted line) and that in IVA blastocysts was expressed relative to one. UD: undetectable. * Sgnificant difference (*p* < 0.05) from FO blastocysts. (**E**,**F**) Confocal images showing the AFIV of ROS and JC-1 staining intensity, respectively, in blastocysts from FO and IVA oocytes. (**F**) Upper and lower images are of the same oocytes observed in TRITC (red fluorescence) and FITC (green) channels, respectively. Bars 140 and 30 µm in panels (**E**,**F**), respectively. Refer to Table S3-3 for numbers of blastocysts used in different graphs.

To find out whether the elevated OS and damaged mitochondria in aged oocyte were transmitted to embryos, ROS and mitochondrial function-related parameters were examined in blastocysts derived from freshly ovulated oocytes or IVA oocytes. While the ROS level was increased (Figure 3A,E), the MMP level was decreased significantly in the IVA- compared to freshly ovulated oocyte-derived blastocysts (Figure 3B,F). Among the four mitochondrial function-supporting genes examined, the mRNA levels of Sirt1, Cox1 and Cox2 were significantly downregulated in IVA oocyte-derived blastocysts compared to those in freshly ovulated oocyte-derived blastocysts (Figure 3C). The mRNA of Pgc1a was undetectable in blastocysts. The mRNA levels of the mitophagy-related genes Pink1 and Parkin were significantly higher in IVA oocyte- than in freshly ovulated oocyte-derived blastocysts (Figure 3D). The results suggest that oocyte IVA increased OS while impairing mitochondrial function in resultant blastocysts.

### 3.8. Effects of IVA on OS and Mitochondria in Hippocampi of F1 Offspring

**Figure 4:**
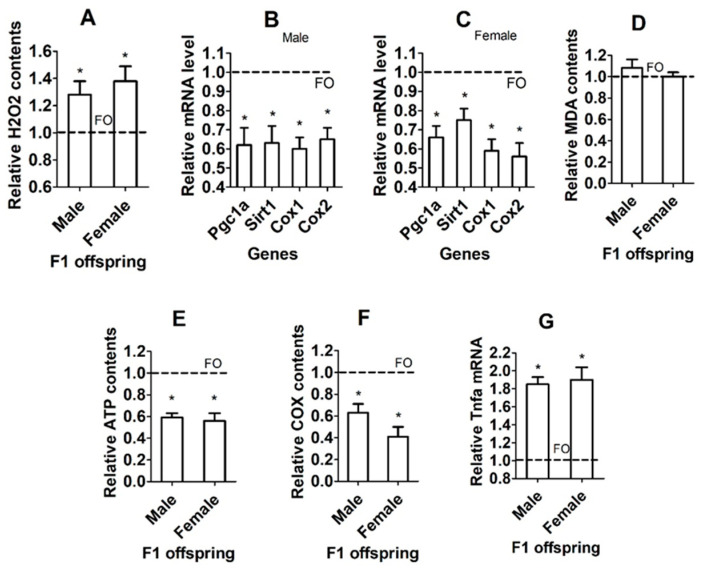
Effects of IVA on OS and mitochondria in hippocampi of F1 offspring. (**A**–**G**) Relative levels of H_2_O_2_, mRNA of mitochondrial function-related genes, MDA, ATP, cytochrome C oxidase (COX), and TNF-α mRNA, respectively, in hippocampi of male and female F1 offspring from freshly ovulated (FO) or IVA oocytes. Each treatment was repeated 6–8 (**A**), 6 (**B**,**C**), 5–6 (**D**,**E**), 4 (**F**) and 5–6 (**G**) times, with each replicate including one mouse from a different litter. The value in FO offspring was set to one and that in IVA offspring was expressed relative to one. * Significant difference (*p* < 0.05) from FO offspring. Refer to Table S3-4 for numbers of mice used in different graphs.

In both male and female offspring, while the level of ROS (represented by H_2_O_2_, Figure 4A) was higher, levels of mitochondrial function-supporting genes (Figure 4B,C), ATP production (Figure 4E) and the cytochrome C oxidase activity (Figure 4F) in hippocampi were significantly lower in IVA oocytes than in freshly ovulated oocyte-derived offspring. The level of malonaldehyde (MDA) did not differ between offspring from freshly ovulated oocytes and those from IVA oocytes (Figure 4D). The level of the inflammatory cytokine TNF-α was significantly higher in IVA- than freshly ovulated oocyte-derived offspring (Figure 4G). The results confirm that oocyte IVA increased OS while impairing mitochondrial function and causing marked inflammation in hippocampi of the resultant F1 offspring. In this experiment, each treatment was repeated four–six times, with each replicate containing one mouse randomly selected from a different litter (Table S3-4). Because the number of F1 mice was high, we used one mouse from each litter to increase the representativeness and to minimize the litter effect.

### 3.9. Effects of ITA in Media with Different Antioxidant Potential on Developmental Potential, OS, and Mitochondria of Oocytes

**Figure 5:**
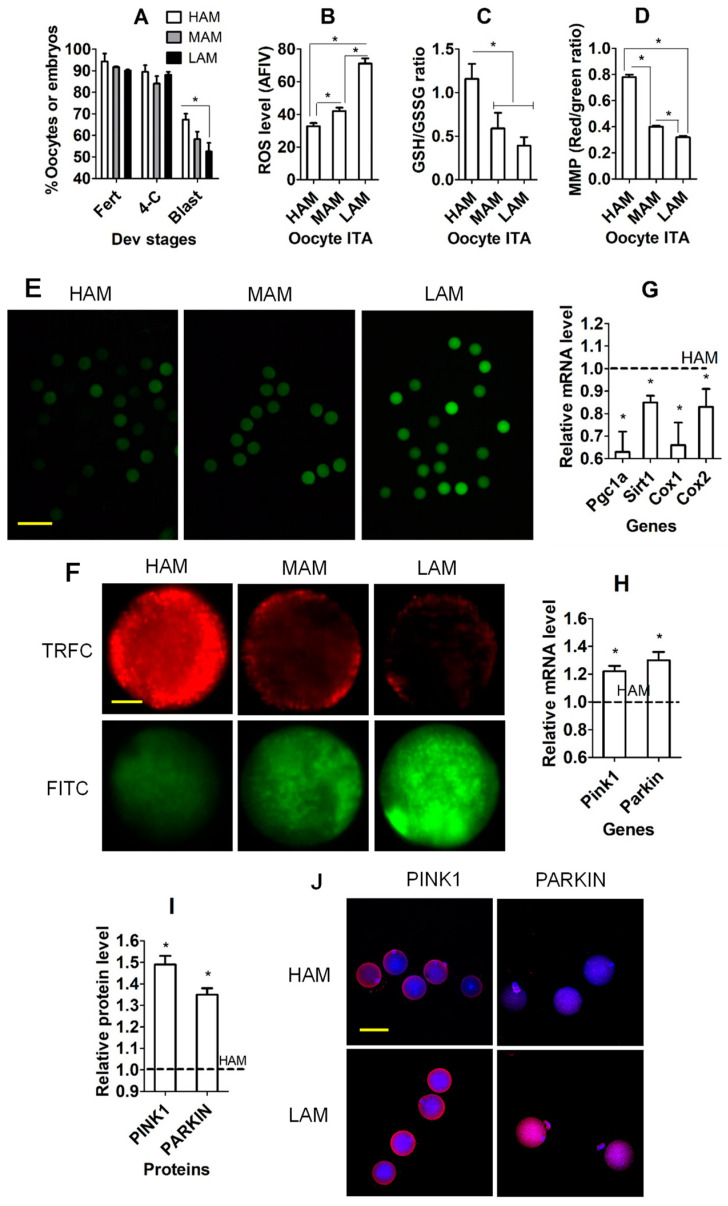
Developmental potential, OS and mitochondria of oocytes after aging in medium with different antioxidant potential. (**A**) Percentages of Fert oocytes and 4-cell and blastocyst embryos after fertilization of oocytes from aging in high (HAM)-, moderate (MAM)-, and low (LAM)-antioxidant medium. Each treatment was repeated 3 times, with each replicate containing 30–35 oocytes. (**B**–**D**,**G**–**I**) Levels of ROS (AFIV), GSH/GSSH ratio, MMP, and mRNA of mitochondria-related genes and mRNA and protein of mitophagy-related genes, respectively, in HAM, MAM, and/or LAM oocytes. Each treatment was repeated 3 times, with each replicate containing about 30 (**B**), 40 (**C**), 25 (**D**), 40–60 (**G**), 40 (**H**), and 20–25 (**I**) oocytes. (**G**–**I**) The mRNA/protein level of HAM oocytes was set to one and that of LAM oocytes was expressed relative to one. * Significant difference (*p* < 0.05). (**E**,**F**,**J**) Confocal images showing ROS, JC-1 and PINK1/PARKIN staining intensity, respectively, in different oocyte groups. (**F**) Upper and lower images are of the same oocytes observed in TRITC (red fluorescence) and FITC (green) channels, respectively. Scale bar 120, 19 and 80 µm in (**E**,**F**,**J**), respectively. Refer to Table S3-5 for numbers of oocytes used in different graphs.

The freshly ovulated oocytes were cultured for 9 h in high-, moderate-, or low-antioxidant medium before fertilization in vitro or examination for levels of ROS, GSH/GSSG ratio, MMP and expression of mitochondria-related genes. Although percentages of fertilized oocytes and four-cell embryos did not differ between high- and low-antioxidant medium, the percentage of blastocysts was significantly lower in low- than in high-antioxidant medium (Figure 5A). While the oocyte level of ROS (Figure 5B,E) was significantly higher, GSH/GSSG ratio (Figure 5C) and MMP (Figure 5D,F) were significantly lower in low- than in high-antioxidant medium. Oocyte levels of the above parameters in the moderate-antioxidant medium oocytes were always between the high- and low-antioxidant medium. Oocyte mRNAs of all four mitochondrial function-supporting genes were downregulated (Figure 5G), and both mRNA (Figure 5H) and protein levels (Figure 5I,J) of PINK1 and PARKIN were upregulated significantly in low- compared to high-antioxidant medium. The results suggest that (a) the oocytes that aged for 9 h in vitro can be fertilized successfully in vitro; (b) compared to aging in high-antioxidant medium, aging in low-antioxidant medium for 9 h significantly increased OS and impaired mitochondrial function of oocytes; and (c) mitophagy increased during early aging as a response to the increase in damaged mitochondria.

### 3.10. Anxiety-Like Behavior and Blood Cortisol Level of F1 Offspring after Oocyte Aging in High-, Moderate-, or Low-Antioxidant Medium

**Figure 6:**
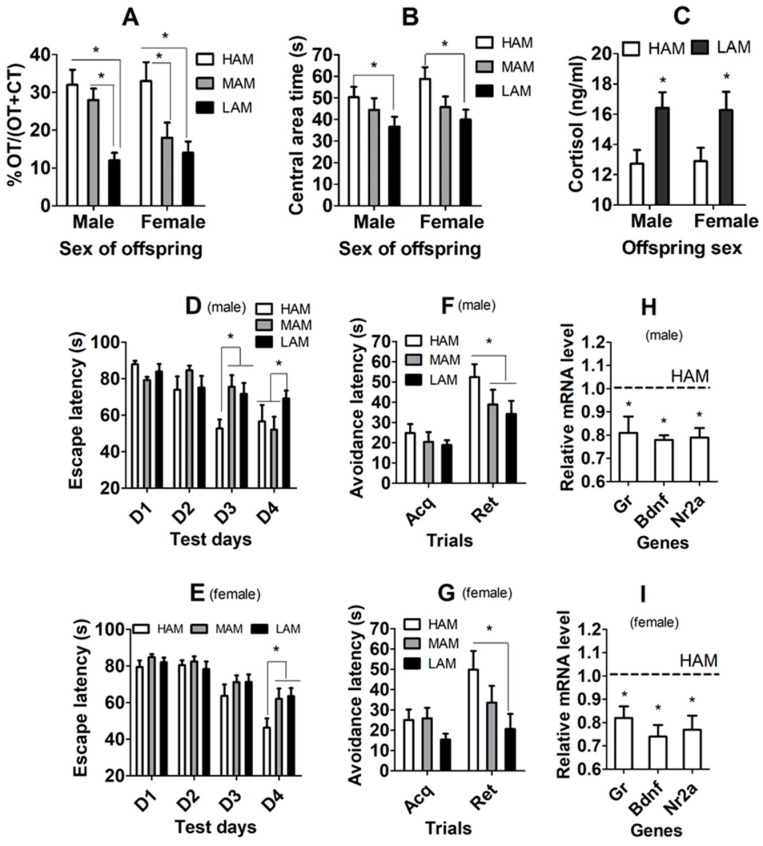
Anxiety-like behavior and spatial/fear learning/memory in F1 offspring after oocyte aging in media with different antioxidant potential. (**A**–**I**) Percentage OT/(OT + CT) of EPM test, times (s) in central area of OFT, serum cortisol concentration, escape latency (s) on different test days in MWM test, latency time (s) of acquisition (Acq) and retention (Ret) trials in PAT, and mRNA levels of Gr, Bdnf and Nr2a genes in hippocampi, respectively, in male and female F1 offspring after transfer of embryos from oocytes aged for 9 h in high (HAM)-, moderate (MAM)-, and low (LAM)-antioxidant medium. For RT-qPCR results, the value in HAM offspring was set to one (dotted line) and the value in LAM offspring was expressed relative to one. For behavioral tests, each treatment contained 16–24 mice from 8–10 recipients. For cortisol measurement, each treatment was repeated 6 times, with each replicate containing serum from one mouse from a different recipient. For RT-qPCR assays, each treatment was repeated 6 times, with each replicate containing one mouse from a different litter. * Significant difference (*p* < 0.05) from HAM oocytes. Refer to Table S3-6 for numbers of mice used in different graphs.

The F1 offspring born from oocytes following aging in high-, moderate-, or low-antioxidant medium were tested for anxiety-like behavior and blood cortisol level 8 and 10 weeks after birth, respectively. In both male and female offspring, while OT/OT + CT during the EPM test (Figure 6A) and the central area time during the OFT (Figure 6B) were shorter, the serum cortisol concentration (Figure 6C) was significantly higher in low-antioxidant medium- than in high-antioxidant medium-derived offspring, with values of the moderate-antioxidant medium-derived offspring in between. The results suggest that oocyte aging in low-antioxidant medium significantly increased the anxiety-like behavior of both male and female F1 offspring.

### 3.11. Spatial/Fear Learning/Memory of F1 Offspring after Oocyte Aging in Medium with High, Moderate or Low Antioxidant Potential

In both male and female offspring, the escape latency was significantly longer on day 4 of the MWM test in low-antioxidant medium-derived offspring than in high-antioxidant medium-derived offspring (Figure 6D,E). On the retention part of the PAT, avoidance latency was significantly shorter in low-antioxidant medium-derived offspring than in high-antioxidant medium-derived offspring (Figure 6F,G). The results suggest that oocyte aging in low-antioxidant medium significantly impaired spatial/fear learning/memory in both male and female F1 offspring.

### 3.12. Levels of Gr, Bdnf and Nr2a mRNA in Hippocampi of F1 Offspring after Oocyte ITA in High- or Low-Antioxidant Medium

The mRNA levels of all the three genes—Gr, Bdnf, and Nr2a—in both male and female offspring were significantly downregulated after aging in low-antioxidant medium compared to aging in high-antioxidant medium (Figure 6H,I). Thus, the results confirm further that ITA in low-antioxidant medium downregulated expression of anxiolytic and learning/memory-beneficial genes.

### 3.13. Effects of ITA in Low-Antioxidant Medium on OS and Mitochondria in Hippocampi of F1 Offspring

**Figure 7:**
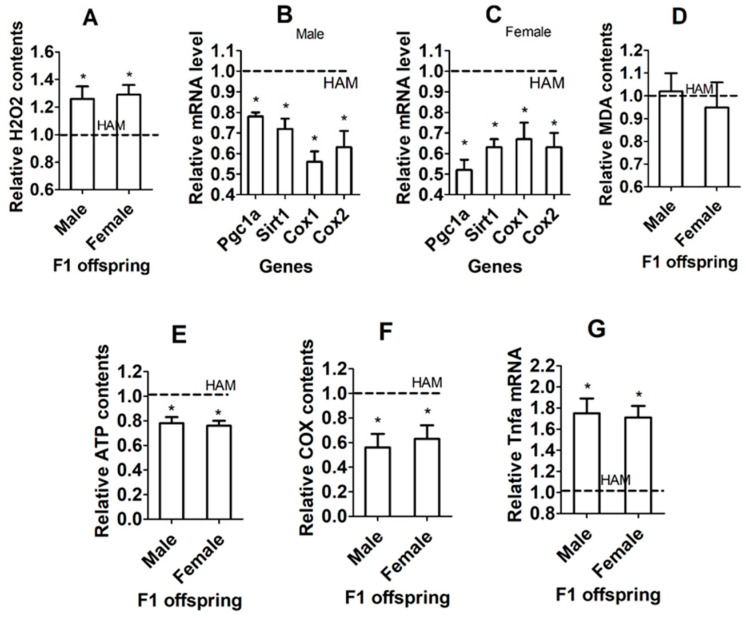
Effects of ITA in low-antioxidant medium on OS and mitochondria in hippocampi of F1 offspring. (**A**–**G**) Relative levels of H_2_O_2_ and mRNA of mitochondrial function-related genes, MDA, ATP, cytochrome C oxidase (COX), and TNF-α mRNA, respectively, in hippocampi of male and female F1 offspring after oocyte aging in high- or low-antioxidant medium. Each treatment was repeated 8 (**A**), 6 (**B**,**C**), 7–8 (**D**), 5–6 (**E**,**G**), and 4–5 (**F**) times, with each replicate including one mouse from a different litter. The value in high-antioxidant medium-derived offspring was set to one and that in low-antioxidant medium-derived offspring was expressed relative to one. * Significant difference (*p* < 0.05) from high-antioxidant medium-derived offspring. Refer to Table S3-7 for numbers of mice used in different graphs.

In both male and female offspring, while the level of ROS (Figure 7A) was higher, levels of mitochondrial function-supporting genes (Figure 7B,C), ATP production (Figure 7E) and cytochrome C oxidase activity (Figure 7F) in hippocampi were significantly lower after oocyte aging in low-antioxidant medium than in high-antioxidant medium. The level of MDA did not differ between high- and low-antioxidant medium (Figure 7D). The level of TNF-α was significantly higher in low- than in high-antioxidant medium (Figure 7G). The results confirm that oocyte ITA increased OS while impairing mitochondrial function and causing marked inflammation in hippocampi of the resultant F1 offspring. In this experiment, each treatment was repeated 4–6 times, with each replicate containing one mouse randomly selected from a different litter (Table S3-7). Because the number of F1 mice was high, we used one mouse from each litter to increase the representativeness and to minimize the litter effect.

### 3.14. Anxiety-Like Behavior and Spatial/Fear Learning/Memory of F2 Offspring from IVA Oocytes

The four types of male or female F2 offspring obtained from mating between freshly ovulated oocyte (FO)- or IVA oocyte-derived F1 and naturally bred (NB) mice were as follows: FO♂ × NB♀, IVA♂ × NB♀, FO♀ × NB♂, and IVA♀ × NB♂. Neither our EPM test (Table S5A) nor the OFT (Table S5B) showed any significant difference in anxiety-like behavior between freshly ovulated oocyte- and IVA oocyte-derived male or female F2 offspring. Neither the MWM (Table S5C) test nor the PAT (Table S5D) revealed any significant difference in spatial/fear learning/memory between freshly ovulated oocyte- and IVA oocyte-derived male or female F2 offspring.

### 3.15. Anxiety-Like Behavior and Spatial/Fear Learning/Memory of F2 Offspring after Oocyte Aging in Low-Antioxidant Medium

The four types of male or female F2 offspring obtained from mating between high (HAM)- or low (LAM)-antioxidant medium-aged oocyte-derived F1 and NB mice were as follows: HAM♂ × NB♀, LAM♂ × NB♀, HAM♀ × NB♂ and LAM♀ × NB♂. Neither our EPM test (Table S6A) nor the OFT (Table S6B) showed any significant difference in anxiety-like behavior between HAM- and LAM-aged oocyte-derived male or female F2 offspring. Neither the MWM (Table S6C) test nor the PAT (Table S6D) revealed any significant difference in spatial/fear learning/memory between HAM- and LAM-aged oocyte-derived male or female F2 offspring.

## 4. Discussion

The present results demonstrate that both IVA and ITA caused significant intra-oocyte OS. Thus, while the level of ROS was higher, the GSH:GSSG ratio was significantly lower in both IVA and low-antioxidant medium-aged oocytes than in control oocytes. Chen et al. [21] also observed a significant increase in ROS level along with a significant decrease in GSH:GSSG ratio during ITA of mouse oocytes. This study also shows that both IVA and low-antioxidant medium aging damaged mitochondria of oocytes. Thus, both the MMP level and the expression of mitochondrial function-supporting genes including Pgc1a, Sirt1, and Cox1/2 were significantly impaired in both IVA and low-antioxidant medium-aged oocytes compared to those in control oocytes. Diminished mitochondrial integrity with a loss of MMP [22] and a decline in ATP production [23] have been observed in aged oocytes. It is known that damage to the mitochondria may cause increased production of ROS [24], which in turn may decrease the GSH:GSSG ratio [25]. Furthermore, decreased expression of Pgc1a [26] and Sirt1 [21] has been observed, though that of Cox1 and 2 has not been reported yet in aged oocytes.

The present study observed OS and mitochondrial dysfunction also in blastocysts and hippocampi of the F1 offspring derived from IVA and low-antioxidant medium-aged oocytes. This suggests that POA caused OS and damaged the mitochondria of oocytes, and the damaged mitochondria entered the adult hippocampi by way of blastocysts carrying information that caused OS and mitochondrial dysfunction and impaired the behavior of F1 offspring. It has been reported that the mitochondria are heavily implicated in virtually every mental or neurological affliction [18], and damaged mitochondria from oocytes can carry information that causes mitochondrial and metabolic dysfunction of the next generation [19]. Furthermore, accumulating evidence demonstrates that OS and mitochondrial dysfunction play an important role in the pathophysiology of neurodegenerative diseases [27]. For example, transplantation of platelet-derived mitochondria into hippocampal neurons can alleviate cognitive impairment and mitochondrial dysfunction in db/db mice [28]. The antioxidant quercetin mitigated methamphetamine-induced anxiety-like behavior through ameliorating mitochondrial dysfunction and neuroinflammation in the hippocampus [29].

It is known that cells maintain a healthy population of mitochondria by degrading damaged mitochondria via mitophagy [30]. However, we observed a significant increase in mitophagy activity along with a significant increase in mitochondrial dysfunction in oocytes and blastocysts after IVA or low-antioxidant medium aging. Boudoures et al. [31] observed that blastocysts generated from high-fat/high-sugar-exposed oocytes had decreased MMP and lower metabolites for ATP generation while showing accumulation of the mitophagy marker protein PINK1. Their explanation was that the increased rate of mitophagy could be a response to the presumed inheritance of damaged mitochondria. When oocytes were observed after IVA for 18 h in this study, expression of mitophagy-related genes became significantly lower than that following IVA for 9 h. Lin et al. [32] observed that during the early stage (up to 12 h) of ITA, autophagy increased as an adaptive response to prevent further apoptosis, but by the late stage (18 h) of ITA, the activation of more caspases blocked the autophagic process, leading to severer apoptosis. Thus, our results suggest that mitophagy increased during early aging as a response to the increase in damaged mitochondria, but this increase was limited by the ongoing apoptosis and thus it failed to eliminate the excessive damaged mitochondria in aged oocytes, leading to their transmission to descendant cells.

The present results show that the F1 offspring from both IVA and low-antioxidant medium-aged oocytes exhibited increased anxiety-like behavior and impaired spatial/fear learning/memory with downregulated hippocampal expression of the Gr, Bdnf and Nr2a genes. Studies have shown that GR, BDNF and NR2A are anxiolytic. For example, both female and male F1 mice from restrained mothers and/or fathers showed significantly reduced anxiety and serum cortisol with increased hippocampal mRNA levels of Gr and Bdnf compared to control offspring from unstressed parents [33]. Both mouse embryo culture with corticosterone and maternal preimplantation restraint stress increased anxiety-like behavior while decreasing hippocampal expression of GR and BDNF in offspring [34]. Both male and female offspring from prenatal stress showed significant anxiety and reduced NR1 and NR2A expression in the hippocampus [35]. Furthermore, naïve female Wistar Kyoto rat offspring displayed an anxiety-like phenotype and a reduction in NMDA receptor subunits in the ventral hippocampus after mothers were treated with fluoxetine during gestation and lactation [36].

Studies have also shown that GR, BDNF and NR2A are learning/memory-beneficial. For example, in utero exposure of mice to diesel exhaust particles (DEPs) affected spatial learning/memory, with reduced hippocampal Nr2a expression in male offspring [37]. Treatment of mice with red wine improved spatial memory and significantly increased mRNA levels of Bdnf and Nr2a in the hippocampus [38]. Overexpression of Gr in the hippocampus attenuated spatial learning and synaptic plasticity deficits after pediatric traumatic brain injury in rats [39]. Furthermore, maternal folic acid supplementation improved learning/memory behavior with Gr upregulation in the hippocampus in mouse offspring [40]. Thus, the current results suggest that oocyte aging with increased OS and mitochondrial dysfunction might affect anxiety-like behavior and learning/memory in offspring by downregulating hippocampal expression of the Gr, Bdnf and Nr2a genes.

In this study, deficits in both spatial and fear learning/memory, which the F1 offspring exhibited during the MWM test and PAT, respectively, were associated with downregulated expression of both Bdnf and Nr2a mRNA in the hippocampus. However, other studies suggested that the fear learning/memory detected by the PAT might not be correlated with expression of the Nr2a gene. For example, Hashikawa-Hobara et al. [38] observed that while treatment of mice with red wine significantly increased hippocampal mRNA levels of both Bdnf and Nr2a, it improved only spatial memory on the MWM test, but advanced the extinction of fear memory on the PAT. Furthermore, Yokota et al. [37] reported that although mice exposed to DEPs in utero showed decreased hippocampal NR2A expression, they exhibited deficits in the MWM test but not the PAT. Thus, POA with increased OS/mitochondrial dysfunction might impair offspring fear learning/memory via pathways differently from those used by other treatments.

The present results show that neither IVA nor low-antioxidant medium aging affected behaviors of F2 offspring. Tarin et al. [2] observed no effect of F0 oocyte aging on the developmental or behavior variables of F2 offspring. While some studies demonstrated that impaired glucose tolerance (IGT) from F1 offspring from gestational diabetes mothers can be transmitted to the F2 but not the F3 generation [41,42], others found that the F2 offspring from oocytes of mothers with pregestational hyperglycemia did not exhibit IGT at all [43]. It is known that exposure of gestating females (F0) to environmental compounds leads to direct exposure of F1 embryo and F2 germ line (primordial germ cells) but not F3, whereas postnatal or adult (F0) exposure results in direct exposure of only F1 germ line (oocytes) but not F2 [44].

## 5. Conclusions

**Figure 8:**
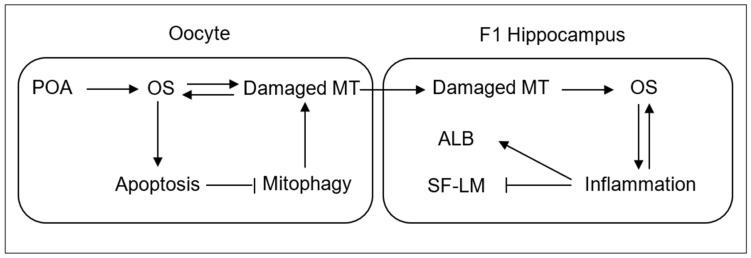
Proposed pathways by which POA increases anxiety-like behavior and impairs spatial/fear learning/memory in F1 offspring. Refer to the last paragraph of Discussion for a detailed explanation.

In conclusion, this study demonstrates that both IVA and low-antioxidant medium aging increased anxiety-like behavior while impairing spatial/fear learning/memory and hippocampal expression of anxiolytic and learning/memory-beneficial genes in both male and female F1 offspring. Furthermore, IVA and low-antioxidant medium aging increased OS with impaired mitochondrial function in oocytes, blastocysts, and hippocampi of F1 offspring. Our results suggest that within the oocyte, POA caused OS, which damaged mitochondria (MT) and triggered apoptosis (Figure 8). Apoptosis has frequently been reported in aged oocytes [21]. The damaged MT would produce more ROS [45] and aggravate OS and apoptosis. The apoptosis would impair mitophagy, leading to the accumulation and transmission to F1 hippocampus of the damaged MT. Within the F1 hippocampus, the damaged MT would increase OS, which would cause inflammation [46]. In turn, inflammation would aggravate OS [45]. It is known that inflammation causes anxiety and depressive disorders [47] and impairs cognition [27]. Together, the data show that POA is a crucial factor that causes psychological problems in offspring, and antioxidant measures may be taken to ameliorate the detrimental effects.

## Data Availability

Data will be made available on request.

## Supplementary Materials

The following supporting information can be downloaded at: https://www.mdpi.com/article/10.3390/cells13090758/s1, Table S1. Gene-specific primers used for real-time PCR; Table S2. Litter size and sex ratio at birth in F1 offspring from freshly-ovulated (FO) or in vivo aged (IVA); Table S3. Numbers of oocytes, blastocysts or mice used in graphs. Table S4. A comparison on behaviors between the F1 offspring and the naturally-bred mice. Table S5. Effects of oocyte in vivo aging (IVA) on anxiety-like behavior (ALB) and spatial/fear learning/memory (SF-LM) in F2 offspring. Table S6. ALB and SF-LM in F2 offspring after oocyte ITA in LAM or HAM.
