# Supplementary material for: Postovulatory Aging of Mouse Oocytes Impairs Offspring Behavior by Causing Oxidative Stress and Damaging Mitochondria
Source: Cells. 2024 Apr 28;13(9):758. doi: 10.3390/cells13090758 (PMC11083947; doi:10.3390/cells13090758)
Supplement: Supplementary file 1 [file cells-13-00758-s001.zip › cells-2961775-supplementary.pdf]

Table S1. Gene-specific primers used for real-time PCR

cDNA	Oligonucleotide sequences (5'-3')	Amplified product size (bp)
Bdnf	F: GCCTCCTCTACTCTTTCTG	255
	R: GGATTACACTTGGTCTCGA	
Gr	F: AGTCAAGGTTTCTGCGT	233
	R: CCATCACTTTTGTTTCG	
Nr2a	F: ACCTCGCTCTGCTCCAGTTTG	131
	R: TCCTGCCATGTTGTCGATGTC	
Pgc1a	F: CCTGCCATTGTTAAGACCGAGAA	142
	R: CTGTGGGTTTGGTGTGAGGAG	
Sirt1	F: ACGGTATCTATGCTCGCCTTG	150
	R: GACACAGAGACGGCTGGAAC	
Cox1	F: CCCTCTATCTACTATTCGGAGCCT	143
	R: AACAAAAGCATGGGCAGTTACGAT	
Cox2	F: CCGAGTCGTTCTGCCAATAGA	144
	R: CGGTTTGATGTTACTGTTGCTTGA	
Pink1	F: GATGACCTTGCAGTTGCTGGAG	141
	R: CAGCAGCCAAAGTCTGAGATCACTA	
Prkin	F: ACCTGCAAACAAGCAACCCT	172
	R: CTACCGACGTGTCCTTGTCT	
Tnfa	F: TATGGCTCAGGGTCCAACTC	126
	R: GGAAAGCCCATTTGAGTCCT	
Gapdh	F: AAGGTGGTGAAGCAGGCAT	244
	R: GGTCCAGGGTTTCTTACTCCT	

Table S2. Litter size and sex ratio at birth in F1 offspring from freshly-ovulated (FO) or in vivo aged (IVA) oocytes

Oocyte treatment	Litter size (Recipients)	Sex ratio (% Males)
FO	6.39 ± 0.54^{a} (18)	51.35^{a} (57/111)
IVA	5.25 ± 0.42^{a} (20)	51.43^{a} (54/105)

a: Values with the same letter in superscripts did not differ ($P>0.05$) between FO and IVA offspring.

Table S3-1. Numbers of oocytes used in figure 1

Graphs	FO	IVA-9h	IVA-18h
A	101	104	
B	104	96	
C	121	120	
D	94	82	
G	180	180	
H	90	90	90
I	PINK1,64; PARKIN,69	PINK1,78; PARKIN,68	

Table S3-2. Numbers of mice (litters) observed in figure 2

Graphs	FO		IVA	
	Male	Female	Male	Female
A	24 (10)	24 (10)	25 (10)	23 (10)
B	26 (10)	24 (10)	26 (10)	26 (10)
C	6 (6)	6 (6)	6 (6)	7 (7)
D	21 (10)		23 (10)	
E		21 (10)		19 (10)
F	21 (9)		21 (10)	
G		20 (10)		21 (10)
H	6 (6)		6 (6)	
I		6 (6)		6 (6)

Table S3-3. Numbers of blastocysts observed in figure 3

Graphs	FO	IVA
A	94	88
B	57	60
C	180	180
D	180	180

Table S3-4. Numbers of mice (litters) observed in figure 4

Graphs	FO		IVA	
	Male	Female	Male	Female
A	6 (6)	6 (6)	6 (6)	6 (6)
B	6 (6)		6 (6)	
C		6 (6)		6 (6)
D	6 (6)	6 (6)	6 (6)	6 (6)
E	6 (6)	6 (6)	6 (6)	6 (6)
F	4 (4)	4 (4)	4 (4)	4 (4)
G	6 (6)	6 (6)	6 (6)	6 (6)

Table S3-5. Numbers of oocytes used in figure 5

Graphs	HAM	MAM	LAM
A	102	104	101
B	106	100	95
C	120	120	120
D	92	90	89
G	180		180
H	180		180
I	Pink1, 69; Parkin, 68		Pink1, 73; Parkin, 63

Table S3-6. Numbers of mice (litters) observed in figure 6

Graphs	HAM		MAM		LAM	
	Male	Female	Male	Female	Male	Female
A	17 (8)	16 (8)	22 (10)	20 (9)	21 (8)	17 (8)
B	21 (8)	21 (8)	24 (10)	20 (9)	17 (8)	17 (8)
C	6 (6)	6 (6)			6 (6)	6 (6)
D	19 (9)		23 (10)		21 (9)	
E		24 (10)		20 (9)		18 (9)
F	21 (9)		24 (10)		19 (9)	
G		22 (10)		20 (9)		17 (9)
H	6 (6)				6 (6)	
I		6 (6)				6 (6)

Table S3-7. Numbers of mice (litters) observed in figure 7

Graphs	HAM		LAM	
	Male	Female	Male	Female
A	6 (6)	6 (6)	6 (6)	6 (6)
B	6 (6)		6 (6)	
C		6 (6)		6 (6)
D	6 (6)	6 (6)	6 (6)	6 (6)
E	6 (6)	6 (6)	6 (6)	6 (6)
F	5 (5)	5 (5)	4 (4)	4 (4)
G	6 (6)	6 (6)	6 (6)	6 (6)

Table S4-1. % Open-arm time (OT)/ (OT + closed arm time (CT)) of EPM in F1 offspring from freshly-ovulated (FO) oocytes and naturally-bred (NB) mice

Offspring sex	Oocyte treatment	Mice observed (Litters)	% OT / (OT+ CT)
Male	NB	22 (8)	0.45±0.07[a]
	FO	22 (8)	0.43±0.06[a]
Female	NB	22 (8)	0.30±0.04[a]
	FO	24 (9)	0.31±0.04[a]

a: Values with the same letter in superscripts did not differ significantly (P>0.05) between FO and NB offspring. (The same for the following tables).

Table S4-2. Time (s) in central area of OFT in F1 offspring from freshly-ovulated (FO) oocytes and naturally-bred (NB) mice

Offspring sex	Oocyte treatment	Mice observed (Litters)	Central time (s)
Male	NB	22 (8)	44.6±4.2[a]
	FO	24 (8)	43.8±4.1[a]
Female	NB	23 (8)	41.3±3.6[a]
	FO	24 (9)	40.0±3.5[a]

Table S4-3. Escape latency (s) on different days in MWM test in F1 offspring from freshly-ovulated (FO) oocytes and naturally-bred (NB) mice

Offspring sex	Treatment	Mice observed (Litters)	Escape latency (s)			
			Day 1	Day 2	Day 3	Day 4
Male	NB	22 (8)	86.8±1.2[a]	72.8±4.7[a]	48.7±4.7[a]	45.9±3.3[a]
	FO	23 (8)	90.0±1.2[a]	66.3±4.6[a]	55.3±4.6[a]	43.4±3.3[a]
Female	NB	22 (8)	84.2±1.8[a]	53.7±4.1[a]	53.0±4.9[a]	41.9±4.7[a]
	FO	24 (9)	86.7±1.7[a]	49.5±4.0[a]	48.5±4.6[a]	44.1±4.4[a]

Table S4-4. The latency time (s) of acquisition (Acq) and retention (Ret) trials in PAT in F1 offspring from freshly-ovulated (FO) oocytes and naturally-bred (NB) mice

Offspring sex	Treatment	Mice observed (Litters)	Avoidance latency (s)	
			Acq	Ret
Male	NB	22 (8)	26.2±3.6[a]	53.3±5.2[a]
	FO	23 (8)	23.6±3.2[a]	52.5±5.1[a]
Female	NB	22 (8)	26.6±2.6[a]	53.7±4.1[a]
	FO	23 (9)	22.6±2.6[a]	49.5±4.0[a]

Table S5. Effects of oocyte in vivo aging (IVA) on anxiety-like behavior (ALB) and spatial/fear learning/memory (SF-LM) in F2 offspring. The male or female F2 offspring were from matings between freshly-ovulated (FO) or 9 h-aged (IVA) oocytes-derived F1 and naturally-bred (NB) mice. The tests were started 8 weeks after birth of the F2 offspring. Table S2A and B show % open-arm time (OT)/OT + closed arm time (CT) of EPM and time (s) in central area of OFT, respectively, in male and female F2 offspring. Table S2C and D show escape latency (s) on different test days in MWM test and the avoidance latency time (s) of acquisition and retention trials in passive avoidance test, respectively, in male or female F2 offspring. a: Values with the same letter in superscripts did not differ significantly (P>0.05) between FO and IVA F2 offspring.

Table S5A. Effects of IVA on ALB of F2 offspring: EPM test

Sex of F2 offspring	F1 mating systems	F2 offspring (Litters)	% OT / (OT+ CT)
Male	FO♂×NB♀	26 (10)	0.35±0.05[a]
	IVA♂×NB♀	21 (9)	0.30±0.06[a]
	FO♀×NB♂	27 (10)	0.29±0.04[a]
	IVA♀×NB♂	27 (10)	0.26±0.04[a]
Female	FO♂×NB♀	24 (8)	0.21±0.04[a]
	IVA♂×NB♀	22 (9)	0.26±0.04[a]
	FO♀×NB♂	23 (9)	0.21±0.03[a]
	IVA♀×NB♂	26 (10)	0.20±0.02[a]

Table S5B. Effects of IVA on ALB of F2 offspring: OF test

Sex of F2 offspring	F1 mating systems	F2 offspring (Litters)	Central area time (s)
Male	FO♂×NB♀	24 (10)	45.60±5.0[a]
	IVA♂×NB♀	20 (9)	43.25±5.4[a]
	FO♀×NB♂	24 (10)	54.86±6.9[a]
	IVA♀×NB♂	23 (9)	54.80±6.9[a]
Female	FO♂×NB♀	22 (8)	53.42±5.3[a]
	IVA♂×NB♀	21 (9)	48.44±5.4[a]
	FO♀×NB♂	19 (10)	56.52±6.6[a]
	IVA♀×NB♂	22 (10)	55.33±6.1[a]

Table S5C. Effects of IVA on spatial memory of F2 offspring: MWM test

Sex of F2 offspring	F1 mating systems	F2 offspring (Litters)	Escape latency (s)			
			Day 1	Day 2	Day 3	Day 4
Male	FO♂×NB♀	24 (9)	89.7±1.1a	68.6±4.4a	50.2±4.4a	44.0±4.4a
	IVA♂×NB♀	23 (9)	87.0±1.1a	71.1±4.3a	55.7±4.4a	45.2±4.4a
	FO♀×NB♂	25 (9)	84.9±2.5a	69.0±4.8a	50.2±4.5a	42.3±3.0a
	IVA♀×NB♂	25 (10)	83.9±2.5a	65.7±4.7a	56.5±4.4a	43.9±2.8a
Female	FO♂×NB♀	23 (9)	84.8±2.3a	66.3±4.4a	53.4±4.7a	43.5±3.7a
	IVA♂×NB♀	24 (9)	82.1±2.2a	66.3±4.3a	52.0±4.7a	41.9±3.7a
	FO♀×NB♂	24 (9)	86.5±1.9a	70.8±4.0a	48.6±4.0a	42.1±4.6a
	IVA♀×NB♂	25 (9)	86.4±1.8a	71.0±3.9a	55.3±3.9a	43.7±4.5a

Table S5D. Effects of IVA on fear memory of F2 offspring: Passive avoidance test

Sex of F2 offspring	F1 mating systems	F2 offspring (Litters)	Avoidance latency (s)	
			Acquisition	Retention
Male	FO♂×NB♀	20 (8)	30.4±2.7[a]	53.3±4.8[a]
	IVA♂×NB♀	20 (8)	22.2±2.8[a]	52.2±4.8[a]
	FO♀×NB♂	23 (8)	27.3±2.9[a]	55.8±4.7[a]
	IVA♀×NB♂	21 (8)	29.2±3.0[a]	51.0±4.9[a]
Female	FO♂×NB♀	22 (8)	22.2±2.6[a]	54.8±4.5[a]
	IVA♂×NB♀	23 (8)	22.7±2.5[a]	52.7±4.4[a]
	FO♀×NB♂	20 (8)	26.1±3.1[a]	53.5±3.8[a]
	IVA♀×NB♂	21 (9)	22.5±3.0[a]	51.7±4.0[a]

Table S6. ALB and SF-LM in F2 offspring after oocyte ITA in LAM or HAM. The male or female F2 offspring were from matings between HAM or LAM oocytes-derived F1 and naturally-bred (NB) mice. The tests were started 8 weeks after birth of the F2 offspring. Tables S3A and B show % OT/(OT+CT) of EPM and times (s) in central area of OFT, respectively, in male and female F2 offspring. Tables C and D show escape latency (s) on different test days in MWM test and the latency time (s) of acquisition and retention trials in passive avoidance test, respectively, in male or female F2 offspring. a: Values with the same letter in superscripts did not differ significantly (P>0.05) between HAM and LAM F2 offspring.

Table S6A. Effects of LAM-ITA on ALB of F2 offspring: EPM test

Sex of F2 offspring	F1 mating systems	F2 offspring (Litters)	% OT / (OT+ CT)
Male	HAM♂×NB♀	25 (9)	0.27±0.06[a]
	LAM♂×NB♀	22 (9)	0.31±0.06[a]
	HAM♀×NB♂	24 (10)	0.31±0.05[a]
	LAM♀×NB♂	22 (9)	0.23±0.05[a]
Female	HAM♂×NB♀	20 (7)	0.31±0.06[a]
	LAM♂×NB♀	20 (7)	0.35±0.05[a]
	HAM♀×NB♂	18 (7)	0.33±0.06[a]
	LAM♀×NB♂	17 (6)	0.27±0.06[a]

Table S6B. Effects of LAM-ITA on ALB of F2 offspring: OF test

Sex of F2 offspring	F1 mating systems	F2 offspring (Litters)	Central area time (s)
Male	HAM♂×NB♀	24 (8)	40.4±4.2[a]
	LAM♂×NB♀	23 (8)	40.8±4.4[a]
	HAM♀×NB♂	21 (8)	50.6±5.2[a]
	LAM♀×NB♂	21 (9)	47.0±5.4[a]
Female	HAM♂×NB♀	21 (8)	49.0±5.5[a]
	LAM♂×NB♀	20 (7)	46.9±5.6[a]
	HAM♀×NB♂	19 (7)	50.4±4.9[a]
	LAM♀×NB♂	21 (7)	50.2±4.6[a]

Table S6C. Effects of LAM-ITA on spatial memory of F2 offspring: MWM test

Sex of F2 offspring	F1 mating systems	F2 offspring (Litters)	Escape latency (s)			
			Day 1	Day 2	Day 3	Day 4
Male	HAM♂×NB♀	25 (9)	89.8±1.8a	70.6±4.4a	51.9±5.8a	46.4±4.0a
	LAM♂×NB♀	23 (8)	84.6±1.9a	69.9±4.6a	55.4±6.0a	44.0±4.3a
	HAM♀×NB♂	23(9)	87.5±1.6a	63.9±4.3a	53.4±3.8a	43.1±3.9a
	LAM♀×NB♂	24 (9)	85.8±1.6a	71.5±4.2a	49.6±3.7a	41.7±3. 8a
Female	HAM♂×NB♀	24 (9)	88.7±1.7a	70.3±3.9a	48.1±3.9a	43.1±4.8a
	LAM♂×NB♀	24 (9)	83.9±1.8a	73.5±4.0a	54.8±a4.0	41.5±4.7a
	HAM♀×NB♂	25 (9)	88.0±1.3a	63.2±4.3a	51.2±4.7a	42.6±4.5a
	LAM♀×NB♂	24 (10)	87.2±1.3a	70.4±4.4a	53.2±4.7 a	41.9±4.6a

Table S6D. Effects of LAM-ITA on fear memory of F2 offspring: Passive avoidance test

Sex of F2 offspring	F1 mating systems	F2 offspring (Litters)	Avoidance latency (s)	
			Acquisition	Retention
Male	HAM♂×NB♀	24 (8)	26.0±3.8[a]	56.6±3.4[a]
	LAM♂×NB♀	22 (8)	23.1±3.7[a]	50.8±3.5[a]
	HAM♀×NB♂	23 (8)	25.6±4.0[a]	56.3±5.6[a]
	LAM♀×NB♂	23 (8)	23.6±4.0[a]	59.1±5.6[a]
Female	HAM♂×NB♀	20 (8)	25.0±2.6[a]	51.7±4.8[a]
	LAM♂×NB♀	22 (8)	25.4±2.6[a]	55.4±4.7[a]
	HAM♀×NB♂	24 (8)	29.2±5.8[a]	50.8±4.3[a]
	LAM♀×NB♂	23 (8)	36.8±5.5[a]	50.4±4. 3[a]
